# Occurrence of Breast Meat Abnormalities and Foot Pad Dermatitis in Light-Size Broiler Chicken Hybrids

**DOI:** 10.3390/ani9100706

**Published:** 2019-09-20

**Authors:** Marco Zampiga, Adele Meluzzi, Stefano Pignata, Federico Sirri

**Affiliations:** Department of Agricultural and Food Sciences, Alma Mater Studiorum—University of Bologna, 40064 Ozzano dell’Emilia (BO), Italy

**Keywords:** broiler chicken, breast meat abnormality, white striping, wooden breast, spaghetti meat, foot pad dermatitis, growth performance

## Abstract

**Simple Summary:**

Limiting the occurrence of breast meat abnormalities and foot pad dermatitis is of vital importance for the overall sustainability of the poultry industry. Although previous findings have revealed that the genotype of the birds could influence the prevalence of both these conditions, only limited information regarding these aspects in current fast-growing broiler genotypes is available. Therefore, this trial was conducted to estimate the incidence and severity of breast myopathies and foot pad dermatitis in two fast-growing chicken hybrids, while simultaneously recording their growth performance. The results obtained in this study showed that the two genotypes, hatched and raised in the same environmental conditions and fed the same commercial diet, showed significantly different occurrence of breast meat abnormalities and foot pad dermatitis, while presenting comparable growth performance at slaughter. This research provides important information that can be useful for both the poultry industry and the scientific community in order to consider the importance of the chicken genotype on crucial meat quality issue and animal welfare aspects such as emerging breast meat abnormalities and foot pad dermatitis, respectively.

**Abstract:**

Only limited information regarding the occurrence of breast meat abnormalities and foot pad dermatitis (FPD) in current broiler genotypes is available. Therefore, this study was conducted to estimate the incidence and severity of breast myopathies (white striping, WS; wooden breast, WB; spaghetti meat, SM) and FPD in two fast-growing chicken hybrids, while simultaneously recording their growth performance. A total of 1560 one-day-old female chicks (780 for each hybrid, A and B; 12 replicates/genotype) were raised in the same environmental conditions and fed the same diet. Productive parameters were recorded at the end of each feeding phase. At slaughter (35 d), the occurrence of meat abnormalities and FPD was assessed on 150 breasts/genotype and on all of the processed birds, respectively. Although comparable growth performance was observed at slaughter, genotype B reported a significantly higher percentage of breasts without meat abnormalities (69% vs. 39%, 75% vs. 41%, 61% vs. 37% for WS, WB and SM, respectively) and also birds without FPD, than genotype A (53% vs. 23%, respectively). Overall, these findings highlight the importance of better understanding the effects of the genotype and the artificial selection applied to fast-growing chicken hybrids on the occurrence of emerging meat abnormalities and FPD even in light-size birds.

## 1. Introduction

Fast-growing broiler chicken hybrids are frequently used in commercial practices to satisfy the increasing consumer demand for poultry meat. These genetic lines can express an extraordinary growth potential, being the result of selective breeding processes aimed at improving vital economic traits for the poultry industry, such as feed efficiency and breast meat yield [1,2,3]. Indeed, fast-growing broiler chickens can be considered as the terrestrial livestock with the highest productive efficiency [4], which is a fundamental aspect of a sustainable agriculture [5]. Conversely, artificial selection for the aforementioned traits in meat-type chickens has been associated with important drawbacks, such as an hyperphagic feeding behavior [6], an increased proneness to obesity [7] and leg disorders [8,9] as well as muscle fibers and meat quality alterations [10,11]. 

In particular, profound modifications of the regular muscle fiber structure may perturb the physiological and biochemical homeostasis within the muscle tissue, possibly leading to a greater occurrence of abnormalities and defects. In recent years, much attention has been given to growth-related muscle abnormalities affecting the *Pectoralis major* muscle of fast-growing broiler chickens such as white striping (WS), wooden breast (WB) and spaghetti meat (SM) defects [12,13,14,15]. Briefly, the WS condition is characterized by the presence of white striations parallel to the muscle fiber direction [16], while breasts presenting the WB abnormality exhibit visible hard, out-bulging and pale areas associated with the possible presence of superficial petechiae and exudate [12]. More recently, the SM defect has been described as an alteration of the structural integrity of the *P. major* muscle which presents an increased tendency of muscle fiber bundles to separate [14,17]. These abnormalities negatively affect nutritional, technological and sensorial traits of breast meat, resulting in remarkable economic losses for the poultry industry [13,15,18]. Although the etiology of these myopathies is still controversial and debated within the scientific community [19,20], a crucial role seems to be played by genetic factors [20]. Indeed, a greater occurrence of muscle abnormalities has been observed in fast-growing, high breast-yield broiler lines [19,20,21,22,23,24], suggesting that genetic strain could be considered one of the major factors involved in the onset of these conditions [15]. 

In addition to meat quality issues, artificial selection for improving performance traits in meat-type chickens is generally associated with a lower resistance towards environmental stressors that can affect overall animal welfare and well-being. In current commercial practices, for instance, meat-type chickens are usually raised by adopting relatively high stocking densities (e.g., 30 to 42 kg of live-weight/m^2^ in EU countries [25]), which have been associated with a poorer litter condition and, in turn, to a greater occurrence of contact dermatitis such as foot pad dermatitis (FPD) [9]. FPD are defined as necrotic lesions affecting the plantar surface of the foot pads in growing broilers and turkeys [26,27]. The presence of these lesions represents a serious issue for the poultry industry from different perspectives. Indeed, not only are FPD considered an important animal welfare indicator worldwide, but they may also negatively affect the economic sustainability of the poultry industry either directly, due to downgrading and condemnation of saleable chicken paws [27], or indirectly, as birds presenting severe FPD could exhibit a pain-induced reduction of growth performance [28,29]. Furthermore, the presence of severe ulcerations in the foot pads could serve as a route for the systemic invasion of microorganisms, with possible implications for animal health and food safety [27]. Although it is widely accepted that environmental conditions such as litter moisture are the main factors influencing the incidence and severity of FPD, previous works have highlighted a different susceptibility in the development of FPD among strain crosses or in fast-growing broiler genotypes commercially available at that time [30,31,32]. 

Despite the huge impact of breast meat abnormalities and FPD on important aspects of the poultry industry, only limited information regarding the occurrence of these conditions in fast-growing genotypes currently used in commercial practices is available, with special regards to light-size birds. To address these concerns, a study was conducted to estimate the incidence and severity of breast myopathies and FPD in two fast-growing chicken hybrids, both currently available and used by the poultry industry, raised in the same environmental conditions and fed the same commercial diet, while also recording, at the same time, their growth performance. 

## 2. Materials and Methods 

All procedures related to handling, raising, and processing of the birds were in compliance with European legislation [25,33,34]. 

### 2.1. Animals, Housing and Diet

A total of 1560 one-day-old female chicks (780 for each hybrid, A and B, respectively) were obtained from a commercial hatchery. The chicken hybrids tested in this trial belong to two different breeding companies and are not genetically related. For each hybrid, eggs were collected from the same breeder flock and then subjected to the same procedures including egg storage, incubation and hatching conditions, as well as chick treatment. After hatching, all chicks were vaccinated according to the plan adopted by the hatchery (coccidiosis, infectious bronchitis virus, Marek’s disease virus, Newcastle and Gumboro disease). The chicks, kept separated according to the genotype, were then housed in an environmental-controlled poultry facility and distributed in 24 floor pens of 6 m^2^ each (12 pens/genotype, 65 birds/pen, 11 birds/m^2^) using chopped straw (2 kg/m^2^) as litter material. Pens were arranged in randomized blocks within the poultry house to limit any environmental effects. Each pen was equipped with two circular pan feeders, able to guarantee at last 2 cm of front space/bird, and 10 nipples. To simulate the environmental conditions usually adopted in commercial practices, the stocking density and photoperiod were defined according to enforced European legislation [25]. Birds received 23L:1D of artificial light during the first 7 d and in the last 3 d of trial, while 18L:6D was used for the remaining period [25]. Both the experimental groups received the same commercial corn–wheat–soybean basal diet (Table 1). A 3-phase feeding program was adopted (starter, 0–9 d, crumbled; grower, 10–21 d and finisher, 22–35 d, pelleted). Diet switches were made uniformly for both of the experimental groups. Water and feed were provided on *ad libitum* basis. 

### 2.2. Productive Performance

The number and weight of the birds were recorded on a pen basis at placement (0 d), at the end of each feeding phase (9 and 21 d) and at slaughter (35 d); then, the body weight (BW) and the daily weight gain (DWG) were calculated accordingly. Feed intake (FI) was measured on a pen basis at the end of each feeding phase (9, 21 and 35 d) and the daily feed intake (DFI) was obtained accordingly. Feed conversion ratio (FCR) was calculated according to these measurements for each feeding phase and for the overall period of the trial. Dead or culled birds were recorded and weighed on a daily basis and their weight was used to correct productive performance data. After 35 d of trial, all birds were processed in a commercial slaughterhouse, applying the same conditions for both the experimental groups (e.g., stunning method, bleeding time, scalding temperature, etc.) as previously described [35]. Similarly, water-bath electrical stunning was used as stunning method, adopting the parameters established by the European Commission [33] (150 mA/bird, 400 Hz). Birds and carcasses belonging to the different experimental groups were clearly identified and kept separated during the processing operations. 

### 2.3. Evaluation of Breast Meat Abnormalities 

The occurrence of WS, WB and SM abnormalities was evaluated on 150 randomly collected breasts/group, approximately 24 h after processing, as previously reported [36,37]. Similarly, a 3-point scale scoring system (NOR: no lesions; MOD: moderate lesions; SEV: severe lesions) was used to classify the severity of each defect. The classification criteria for WS, WB, and SM defects were, respectively, the dimension of white striations [38], the hardness at palpation [12], and the proneness to show muscle deconstruction in response to finger pinching [17]. All of the evaluations were performed by the same well-trained operator in analogous environmental conditions. 

### 2.4. Evaluation of Foot Pad Dermatitis

A foot from each bird was systematically collected during processing operations and subjected to a macroscopic evaluation of the necrotic lesions affecting the ventral surface of the foot pad. The severity of FPD was classified into three categories according to the scoring procedure proposed by Ekstrand et al. [39]: 0—no lesion; 1—mild lesion (diameter <0.8 cm); and 2—severe lesion (diameter >0.8 cm). In addition, the lesion score was calculated according to the formula reported in our previous study [40]. Briefly, the number of feet belonging to class 1 and 2 was multiplied by 0.5 and 2, respectively. The scores were then added, and the total was divided by the number of collected samples and multiplied by 100. The number of feet in class 0 did not contribute to the score. 

### 2.5. Statistical Analysis

Productive performance data were analyzed through the Student *t*-test, considering the genotype of the birds as an independent variable [41]. The effect of the pen was not statistically significant for each trait; hence, it was not included in the statistical model. Chi-square test was used to evaluate the frequency distribution of the severity classes of breast meat abnormalities and FPD [41]. Mortality percentage was submitted to arcsine transformation prior to analysis. The experimental unit was the pen for productive performance data, while the single bird was considered as the experimental unit for the occurrence of breast myopathies and FPD. 

## 3. Results

### 3.1. Productive Performance 

The productive performance of both the hybrids in each feeding phase and in the overall period of trial is shown in Table 2. At placement, chicks belonging to group A were heavier than the counterpart (44.1 vs. 41.0 g, for A and B, respectively; *p* < 0.01). After 9 d, A birds maintained higher BW than B ones (227 vs. 216 g, respectively; *p* < 0.01). Similarly, DWG was greater in the A group (20.3 vs. 19.4 g/bird/d for A and B, respectively; *p* < 0.05). No significant effect was observed on DFI, FCR and mortality. From 10 to 21 d, birds in the A group exhibited lower BW and DWG in respect to the B group (751 vs. 775 g and 43.5 vs. 46.6 g/bird/d, respectively; *p* < 0.01). Although no significant effect was observed on DFI, FCR was significantly higher in group A than in group B (1.632 vs. 1.511, respectively; *p* < 0.05). Mortality was not significantly affected by the chicken genotype. During the finisher phase (22 to 35 d), BW, DWG, FCR and mortality were similar between the groups, although group A showed lower DFI (138.1 vs. 142.5 g/bird/d for A and B, respectively; *p* < 0.05). Considering the overall period of the trial (0 to 35 d), the genotypes exhibited comparable BW, DWG, DFI, FCR and mortality resulting in no significant difference in terms of overall productive performance. At slaughter, carcass yields were respectively 67.3% vs. 68.8% for A and B.

### 3.2. Incidence and Severity of Breast Myopathies 

**Figure 1:**
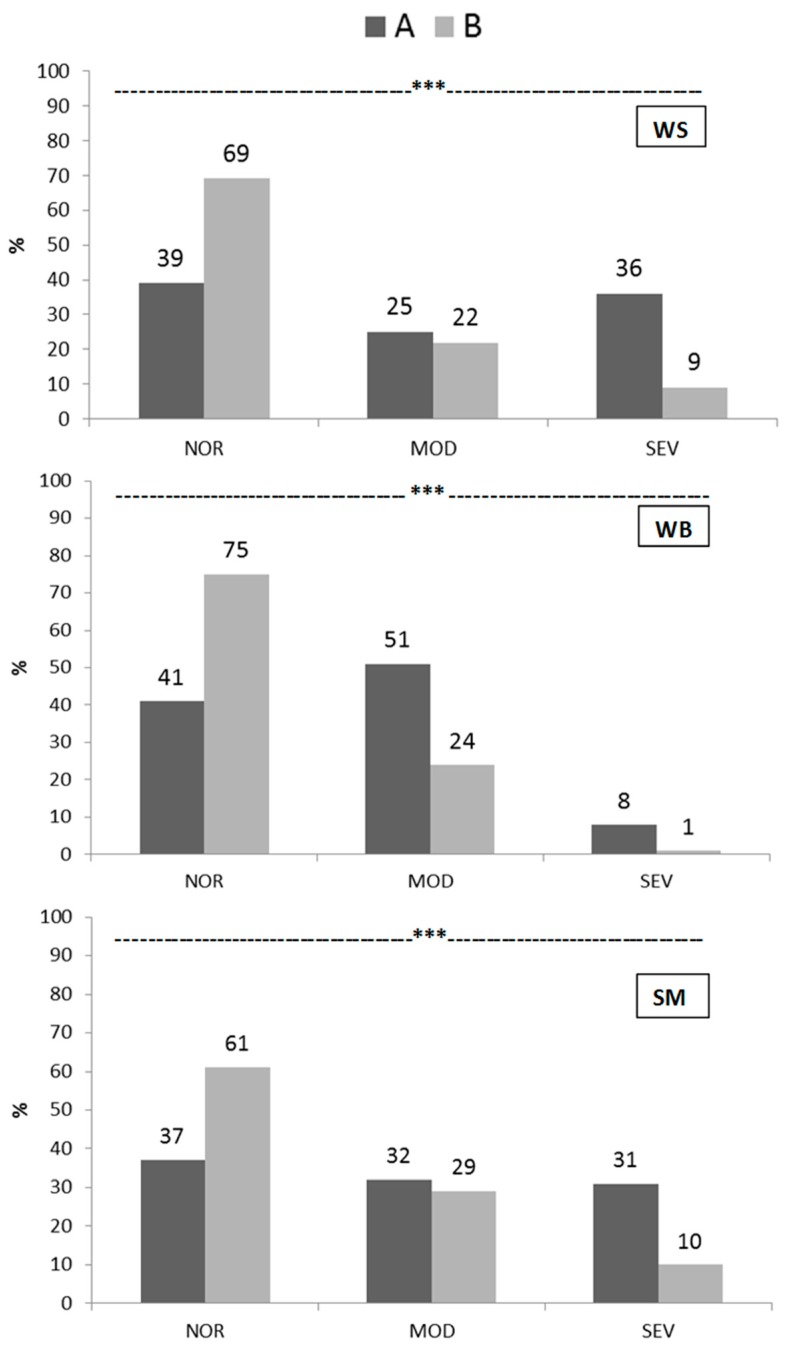
Incidence (%) and severity (no lesions (NOR), moderate lesions (MOD) and severe lesions (SEV)) of white striping (WS), wooden breast (WB) and spaghetti meat (SM) defects in both genotypes (A and B) at slaughtering (35 d) (*** *p* < 0.001).

The incidence and severity of WS, WB and SM abnormalities in both genotypes are depicted in Figure 1. Regarding WS, genotype A reported a lower percentage of breasts with no lesions (39% vs. 69%, respectively for A and B; *p* < 0.001) coupled with a higher percentage of those showing severe lesions (36% vs. 9%, respectively for A and B; *p* < 0.001). Similarly, genotype A was characterized by a lower percentage of breasts presenting no WB lesions (41% vs. 75%, respectively for A and B; *p* < 0.001) while the occurrence of moderate and severe WB cases was higher (51% vs. 24% and 8% vs. 1%, respectively for A and B; *p* < 0.001). Finally, if compared to group B, group A reported a lower percentage of breasts with no SM defect (37% vs. 61%, respectively for A and B; *p* < 0.001) associated with a higher incidence of severe SM cases (31% vs. 10%, respectively for A and B; *p* < 0.001). 

### 3.3. Incidence and Severity of Foot Pad Dermatitis

**Figure 2:**
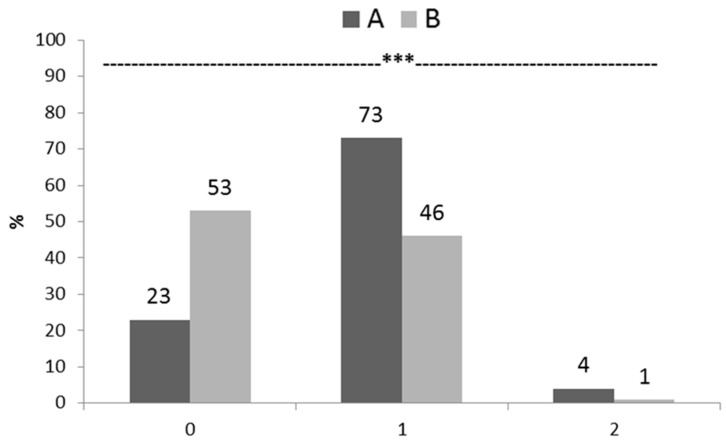
Incidence (%) and severity (0: no lesion; 1: mild lesion; 2: severe lesion) of foot pad dermatitis in both genotypes (A and B) at slaughtering (35 d) (*** *p* < 0.001).

In Figure 2, the incidence and severity of FPD are reported. Genotype A was characterized by a lower percentage of birds presenting no lesions (23% vs. 53%, respectively for A and B; *p* < 0.001) as well as a higher percentage of those with moderate and severe necrotic lesions (73% vs. 46% and 4% vs. 1% respectively for A and B; *p* < 0.001). According to these results, the lesion score was 44.3 and 25.0 in A and B groups, respectively, indicating an overall better condition of the foot pads for the birds belonging to group B.

## 4. Discussion

Breast meat abnormalities can be considered the major meat quality issue currently faced by the poultry industry. The economic impact of these meat aberrations in the U.S. poultry sector has been estimated to be around $200 million per year (conservative estimate) due to processing yield reduction (e.g., trimming, drip loss, cook loss) as well as increased meat downgrading or discarding rate [13]. Furthermore, the necrotic and ulcerative lesions associated with FPD represent an important animal welfare indicator [9]. Additionally, FPD can be considered a food safety and product quality issue as well. Indeed, Shepherd and Fairchild [27] reported that the demand for high-quality paws in export markets (mainly China and Hong Kong) has been increasing since the 1980s, turning chicken paws into the third most important economic part of the chicken carcass (approximately $280 million/year [42]). Therefore, it is fundamental to assess whether the incidence and severity of breast meat abnormalities and FPD could vary among the commercial broiler chicken genotypes that are currently available and used by the poultry industry. It should be noted that these conditions can be widely affected by a plethora of different environmental factors that need to be carefully controlled in order to discern which is the real effect of the chicken genotype on breast meat abnormalities and FPD occurrence. In this study, for each genotype, fertile eggs were obtained from the same breeder flock and subjected to the same incubation procedures. Then, the birds were raised in the same environmental conditions, fed the same commercial diet throughout the trial and finally processed in a commercial slaughterhouse applying the same procedures. Therefore, any environmental effects could be reasonably excluded from the experimental model.

According to the results obtained in this study, it emerged that broiler chickens belonging to the studied genotypes showed a significantly different magnitude of breast meat abnormalities and FPD, while achieving similar growth performance at slaughter. Indeed, although genotype A showed a higher chick weight and greater precocity, birds belonging to the hybrid B exhibited higher BW and DGW, coupled with a lower FCR, from 10 to 21 d, resulting in a comparable performance for the entire rearing cycle. Overall, these growth patterns confirmed those observed in our previous study, wherein female chickens of the same genotypes were raised until 43 d [43].

Considering breast meat abnormalities, genotype B reported a lower incidence and severity of WS, WB and SM defects, suggesting that this genetic line is less prone to developing breast myopathies, as compared to genotype A, at 35 d of age. Indeed, more than 60% of the breasts belonging to genotype B did not exhibit any visible sign of either WS, WB or WS conditions, while the percentage of those presenting severe lesions was lower than 10% for each defect. Furthermore, these data demonstrated the presence of these meat quality defects even in light-size female birds slaughtered at a relatively early age (35 d), which is an area of study that typically receives limited attention, as it is generally recognized that light-size chickens are less prone to develop such degenerative alterations as compared to heavy broilers. Taken together, these results seem to confirm the significant effect of the genotype on the development of breast meat abnormalities in broiler chickens. Although the reasons behind the different magnitudes of muscle myopathies in the tested hybrids are still unknown, future studies regarding the genetic basis of these birds, as well as the effect of the different growth patterns, may provide insightful information on this topic. Limiting the occurrence of breast meat abnormalities is of vital importance to the economic sustainability of the poultry industry. As nutritional treatments showed only mild efficacy, as reviewed by Petracci et al. [15], the choice of genotypes exhibiting a lower tendency toward breast myopathies might represent a valuable strategy for poultry producers in achieving this result. To our knowledge, only limited information regarding the occurrence of breast muscle myopathies in current commercial broiler genotypes is available. Bauermeister et al. [44] reported significant differences in the occurrence and severity of WS in two fast-growing strains of male broilers, processed at 56 d of age, showing different fillet weight. On the other hand, a significant effect of the genotype on the occurrence of WS defect was also demonstrated in male turkeys [45]. Alnahhas et al. [20] pointed out that the role of genetics could be considered as a major determinant of the WS condition in broilers. Indeed, it is generally recognized that high-breast yield broiler strains are more prone to develop the WS abnormality, as compared to standard-breast yield strains [19,20,21,22,23]. Moreover, the occurrence and severity of WB were also reported to be significantly affected by the genetic background of the broilers [24]. However, to the best of our knowledge, no information regarding the occurrence of the SM abnormality in different fast-growing broiler hybrids is currently available.

As for FPD occurrence, genotype B exhibited an overall better condition of the foot pads compared to genotype A one when raised on fresh chopped straw litter, as indicated by the higher percentage of birds presenting with no lesions and, consequently, by the lower lesion score. In general, the poultry industry has a vested interest in producing unblemished paws, both in terms of complying with animal welfare recommendations and for increasing profitability, as the price of high-quality paws has increased significantly due to the large export market in Asia [27]. Therefore, the identification of genetic strains of broiler chickens characterized by a greater foot pads resistance toward the challenging environmental conditions currently faced in intensive systems, but also in alternative ones, is a key point for preserving animal welfare and profitability. Similar to our findings, Kestin et al. [30] reported differences in FPD scores among four different crosses, revealing a possible different susceptibility to developing foot pad lesions according to the genotype. Likewise, Bilgili et al. [32], investigating the effect of strain-cross on the development of FPD along with diet densities, found a significant interaction at 42 d of age and concluded that the susceptibility to FPD may vary by strain-cross. A similar conclusion has been drawn by Kjaer et al. [46] who reported that Ross 308 broilers exhibited a higher occurrence of FPD in comparison to a slow-growing, dual-purpose strain. Finally, a significantly lower prevalence of FPD was detected in Swedish Cobb chicks than in either Danish or Swedish Ross ones, although the different body weights and housing conditions could have influenced these findings, as declared by the authors [31].

## 5. Conclusions

In conclusion, the results obtained in this study revealed that broiler chickens belonging to two different genotypes, hatched and raised in the same environmental conditions and fed the same commercial diet, showed significantly different occurrences of breast meat abnormalities and FPD at 35 d of age, while presenting comparable growth performances at slaughter. These findings highlight the importance of better understanding the effects of the genotype and the artificial selection applied to fast-growing chicken hybrids on the occurrence of the main emerging meat abnormalities, which negatively affect the economic sustainability of the poultry industry, as well as on important animal welfare indicators, such as foot pad dermatitis, even in light size birds.

## Figures and Tables

**Table 1:** Composition of the basal diet administered to both A and B hybrids in each feeding phase.

	Starter 0–9 d	Grower 10–21 d	Finisher 22–35 d
Ingredients, g/100 g			
Corn	33.4	36.7	34.2
Wheat	20.0	20.0	25.0
Vegetable oil	2.45	2.68	3.61
Soybean meal 48%	18.2	20.2	14.2
Full-fat soybean	10.0	10.0	15.0
High-protein soybean meal	5.00	0.00	0.00
Sunflower	2.00	2.00	2.00
Pea	3.00	3.00	3.00
Corn gluten	2.00	2.00	0.00
Lysine	0.54	0.53	0.46
DL-Methionine	0.29	0.32	0.33
L-Threonine	0.12	0.11	0.10
Choline chloride	0.10	0.10	0.05
Calcium carbonate	0.53	0.52	0.60
Dicalcium phosphate	1.29	0.80	0.47
Sodium chloride	0.29	0.30	0.23
Sodium bicarbonate	0.05	0.05	0.15
Vit.-min premix ^1^	0.54	0.46	0.38
Phytase	0.05	0.05	0.05
Xylanase	0.05	0.05	0.05
Emulsifier	0.08	0.08	0.08
Calculated chemical composition			
Dry matter *, %	88.8	88.2	88.5
Crude protein *, %	22.7	21.0	19.1
Total lipid *, %	6.25	6.51	8.29
Crude fiber *, %	2.96	2.92	2.99
Ash, %	5.24	4.60	4.29
Lysine (total), %	1.42	1.31	1.20
Met. + Cyst. (total), %	0.99	0.92	0.85
Arginine (total), %	1.46	1.34	1.25
Threonine (total), %	0.94	0.87	0.79
Ca (total), %	0.77	0.62	0.55
P (total), %	0.61	0.51	0.44
AME, kcal/kg	3100	3150	3275

^1^ Provided the following per kg of diet: vitamin A (retinyl acetate), 13,000 IU; vitamin D3 (cholecalciferol), 4000 IU; vitamin E (DL-α_tocopheryl acetate), 80 IU; vitamin K (menadione sodium bisulfite), 3 mg; riboflavin, 6.0 mg; pantothenic acid, 6.0 mg; niacin, 20 mg; pyridoxine, 2 mg; folic acid, 0.5 mg; biotin, 0.10 mg; thiamine, 2.5 mg; vitamin B12 20 μg; Mn, 100 mg; Zn, 85 mg; Fe, 30 mg; Cu, 10 mg; I, 1.5 mg; Se, 0.2 mg; ethoxyquin, 100 mg. * Analyzed values.

**Table 2:** Productive performance of broiler chickens of both the genotypes (A and B) in each feeding phase and in the overall period of trial.

Variables	A	B	SEM	*p*-Value
n. replicates	12	12		
0–9 d				
Chick body weight (g/bird)	44.1 ^A^	41.0 ^B^	0.38	<0.01
Body weight (g/bird)	227 ^A^	216 ^B^	2.32	<0.01
Daily weight gain (g/bird/d) *	20.3 ^a^	19.4 ^b^	0.26	<0.05
Daily feed intake (g/bird/d) *	25.1	24.0	0.53	ns
Feed conversion ratio *	1.235	1.237	0.02	ns
Mortality (%)	0.12	0.00	0.01	ns
10–21 d				
Body weight (g/bird)	751 ^B^	775 ^A^	5.77	<0.01
Daily weight gain (g/bird/d) *	43.5 ^B^	46.6 ^A^	0.57	<0.01
Daily feed intake (g/bird/d) *	71.0	70.3	1.50	ns
Feed conversion ratio *	1.632 ^a^	1.511 ^b^	0.04	<0.05
Mortality (%)	0.31	1.15	0.02	ns
22–35 d				
Body weight (g/bird)	1929	1962	13.8	ns
Daily weight gain (g/bird/d) *	84.1	84.8	0.88	ns
Daily feed intake (g/bird/d) *	138.1 ^b^	142.5 ^a^	1.20	<0.05
Feed conversion ratio *	1.643	1.681	0.02	ns
Mortality (%)	0.31	0.10	0.01	ns
0–35 d				
Body weight (g/bird)	1929	1962	13.8	ns
Daily weight gain (g/bird/d) *	53.8	54.9	0.39	ns
Daily feed intake (g/bird/d) *	86.0	87.0	0.68	ns
Feed conversion ratio *	1.600	1.590	0.01	ns
Mortality (%)	0.85	1.25	0.02	ns

* corrected for mortality. Means within a row not sharing a common superscript are significantly different (A, B: *p* < 0.01; a, b: *p* < 0.05). SEM: standard error mean. ns: not significant.
